# (Dis)Trust your gut: the gut microbiome in age-related inflammation, health, and disease

**DOI:** 10.1186/s40168-017-0296-0

**Published:** 2017-07-14

**Authors:** Thomas W. Buford

**Affiliations:** Department of Medicine, University of Alabama at Birmingham, 933 19th Street South, Birmingham, AL 35294 USA

## Abstract

Chronic inflammation represents one of the most consistent biologic features of aging. However, the precise etiology of persistent low-grade increases in inflammation remains unclear. Recent evidence suggests that the gut microbiome may play a key role in age-related inflammation. Indeed, several studies have indicated that older adults display an altered composition of the gut microbiota, and early evidence indicates that this dysbiosis is associated with the presence of several key circulating inflammatory analytes. The present review summarizes knowledge on age-related inflammation and discusses how potential relationships with gut dysbiosis may lead to novel treatment strategies in the future.

“The pattern of disease is an expression of the response of man to his total environment (physical, biological, and social); this response is, therefore, determined by anything that affects man himself or his environment.” – Rene Dubos, 1961

## Background

In humans, aging is a continual and progressive process that results in decreased physiologic function across all organ systems [1]. These physiologic decrements result in an increased vulnerability to infection and disease that dramatically elevates mortality risk [2, 3]. Compared to persons 25–44 years of age, mortality risk among older adults is elevated by 100-fold for stroke and chronic lung disease, roughly 90-fold for heart disease, pneumonia, and influenza, and over 40-fold for cancer [2]. Given the rapid increase in life expectancy and increasing proportion of older adults within the population [4], an increased understanding of the biologic mechanisms which underlie age-related increases in disease prevalence are warranted.

## Aging and inflammation

**Fig. 1:**
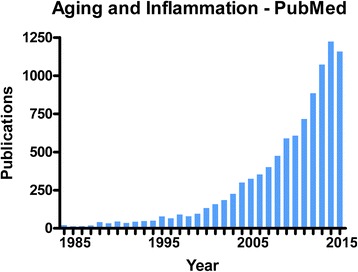
Number of PubMed citations by year using the search term “aging and inflammation”

**Fig. 2:**
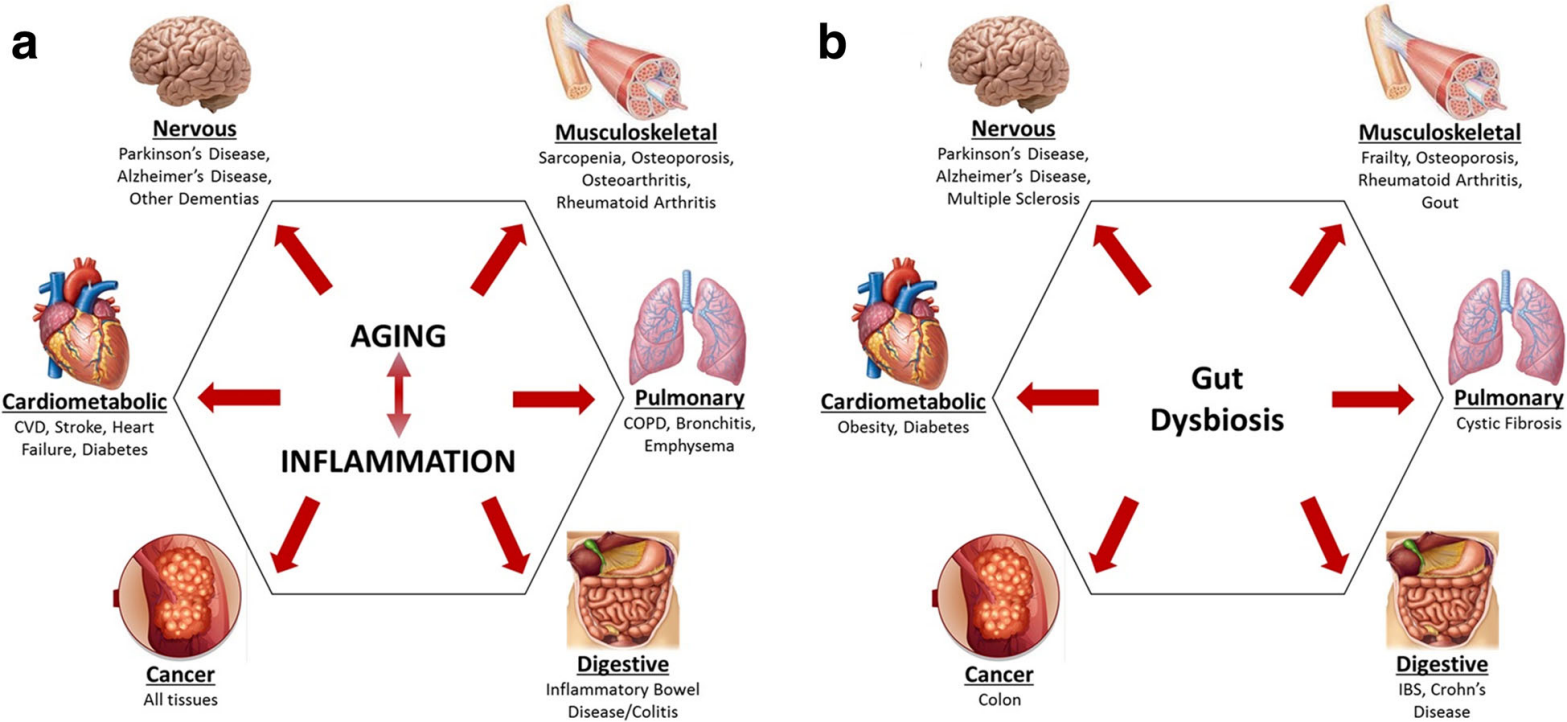
**a** Prominent health conditions with both biologic age and chronic inflammation as central risk factors. **b** Prominent health conditions with evidence linking them to gut dysbiosis. Note the similarities between the conditions associated with aging and inflammation and those associated with gut dysbiosis

Though the etiologies of age-related diseases are quite diverse, significant evidence implicates chronic, low-grade inflammation as one of the most consistent biologic features of both chronological aging and various age-related diseases/disorders [5–8]. In fact, a recent PubMed search for the terms “aging and inflammation” revealed nearly 10000 publications in this area (Fig. 1) with this pair of risk factors implicated in the pathobiology of a wide variety of diseases and disorders across nearly every organ system (Fig. 2a).

Inflammation is commonly recognized as a localized response to tissue injury or infection that aids in the repair of damaged tissue and/or destruction of the harmful agent [4]. Classically characterized by pain, heat, redness, swelling, and loss of function, acute inflammation is typically resolved (in healthy individuals) in relatively short order to promote the restoration of tissue function. However, during advanced age, the ability to resolve inflammation becomes impaired leading to sustained tissue infiltration of leukocytes and the chronic release of pro-inflammatory cytokines and chemokines [9]. As a result, the initial local event has long-term systemic consequences.

Indeed, elevations in pro-inflammatory mediators such as interleukin-6 (IL-6), C-reactive protein (CRP), and tumor necrosis factor alpha (TNF-α) have been consistently reported during late adulthood even in the absence of acute infection [10–12]. Though it is unknown if these inflammatory mediators directly cause adverse health outcomes, chronic low-grade inflammation has been associated with the prevalence of a wide-range of age-related co-morbidities—cardiovascular disease [13–15], insulin resistance and diabetes [16–18], osteoporosis [19–21], cognitive decline and dementia [22–24], frailty and disability [25–27], and cancer [28–30]—as well as mortality [31–33].

Though factors such as obesity and insulin resistance, smoking, and changes in circulating sex hormone concentrations have been associated with age-related inflammation [34], the largest contributor to increases in inflammatory mediators has traditionally been thought to decrease in the efficiency of the immune system, i.e., immuno-senescence [6, 8]. Immuno-senescence is characterized by thymus atrophy, reductions in neutrophil function, naïve T cell number, and the cytotoxic capacity of natural killer cells, and lowered B-cell antibody production in response to antigen [35, 36]. The most widely held belief regarding the cause of immune-senescence is that chronic lifetime antigenic burden exhausts a finite capacity of the organism’s immune system [4, 37, 38]. Therefore, we exchange protection against infection for long-term risks of chronic inflammation and disease [4, 39].

Very recently, however, emerging evidence has suggested that the gut microbiome may play an integral role in these age-related inflammatory changes. Recent evidence indicates that advanced age is associated with changes in microbiota composition characterized by a loss of diversity in the core taxa [40]. Moreover, these age-related changes in the microbiome have also been recently associated with key geriatric syndromes including physical frailty [41] and dementia [42]. Therefore, the objective of this review is to discuss the available evidence related to gut microbiome and aging, with particular emphasis on age-related inflammation and associated diseases/conditions.

## The gut microbiome and health

The human intestinal tract (i.e., “gut”) is inhabited by over 100 trillion microorganisms; including over 1000 species of known bacteria.[43] These organisms have co-evolved with humans over millennia to live together for mutual benefit [44, 45]. Though commonly overlooked in considerations of human health and disease treatment (hence the nickname the “forgotten organ” [46]), gut microorganisms encode >150 times more genes than the human genome and are highly involved in numerous metabolic reactions which influence normal host physiology and metabolism [47, 48]. In fact, an enormous portion of the body’s constitutive immune function is dedicated to maintaining homeostasis with the microbiota, as evidenced by the fact that 70% of the body’s lymphocytes reside in the gut-associated lymphoid tissue [49].

Given the substantial portion of the immune system dedicated to maintaining host relationship with the microbiome, it is perhaps unsurprising that gut microbiota are heavily involved in local inflammatory responses to acute injury and/or infection. Indeed, commensal bacteria are a critical regulator of regulatory immune responses including tolerance—the active suppression of inflammatory responses to food and other orally ingested antigens [50]. In fact, these bacteria are known to play a protective role in acute inflammatory responses to injury at least in part through toll-like receptor (TLR) activation to promote tissue repair and survival [51]. Moreover, immune cells also regulate inflammation in intestinal injury and allergy via recognition of short-chain fatty acids (SCFA) produced via bacterial breakdown of indigestible dietary components such as fiber [52].

Failure to regulate these responses is well-established as a contributor to the development of food allergies and/or various inflammatory bowel diseases affecting the gut. More recently, however, it has become apparent that changes to microbial composition and density can alter immunity and inflammation within organs distal from the intestine [53]. For example, the reduction of beneficial gut bacteria via antibiotic treatment has demonstrated impaired systemic allergic inflammatory responses as well as immune responses to viral (influenza) infections [54–56]. Though beyond the scope of the current review, I refer readers to an excellent review by Belkaid and Hand [50] for in-depth discussion of the potential mechanisms underlying microbiota-based changes to systemic immunity and inflammation.

In line with known interactions of the microbiome with the systemic circulation and distal tissues, gut dysbiosis—commonly defined as a disturbance of or change in the density and/or composition of gut microbiota—has been linked to a wide variety of diseases and health conditions (Fig. 2b). These conditions include cystic fibrosis [57], inflammatory bowel conditions (irritable bowel syndrome, Crohn’s disease, and colon cancer [58–60], neurological diseases (Parkinson’s disease, Alzheimer’s disease, and multiple sclerosis)) [61–63], metabolic diseases (obesity and diabetes) [64, 65], as well as musculoskeletal conditions (frailty, osteoporosis, rheumatoid arthritis, and gout) [41, 66–68]. Notably, as evidenced by the remarkable similarity between Fig. 2a, b, the majority of these conditions exhibit age-related increases in incidence. Accordingly, it seems relevant to explore the possibility that age-related inflammation may stem at least partially from changes to the gut microbiome.

## The aging gut microbiome

As noted above, the human ageing process is associated with gradual declines in function across virtually every bodily organ. In contrast, however, the bacterial organisms in the gut do not age per se and thus might be unexpected to follow the typical trajectory of physiological decline seen elsewhere in the body [69]. And yet, as pointed out by O’Toole and Jeffery [69], older individuals often experience gut-associated comorbidities, changes in diet and physical activity patterns, and other gut-related physiologic changes which may influence gut bacteria. Thus, questions persist regarding how such changes may interact with gut bacteria to influence microbiome composition and function.

Compared to evidence related to aging and inflammation, less is known regarding associations between aging and the microbiome. In fact, in contrast to the thousands of peer-reviewed publications on aging and inflammation, a PubMed search for “aging and microbiome” yielded only 466 results and a search for “aging and dysbiosis” yielded a mere 34. Moreover, only a handful of studies to date have investigated the aging microbiome in humans (Table 1). Still, at least two early studies in this area have documented that advanced age is associated with changes to both the composition and stability of gut microbiota [40, 70]. Biagi et al. reported that a group of centenarian from Northern Italy displayed low species diversity compared to younger adults (~30 years of age). They also noted specific changes within Firmicutes (one of the two dominant phyla commonly found in the gut) subgroups and enrichment of Proteobacteria—a group containing many opportunistic bacteria which can overtake commensal bacteria and induce pathology [70]. These microbiome changes were also characterized by a loss of genes for short-chain fatty acid production and an overall decrease in the saccharolytic potential, while proteolytic functions were more abundant than in the intestinal metagenome of younger adults [71]. Interestingly, these changes in bacterial content were also moderately associated with circulating plasma concentrations of inflammatory cytokines interleukins six (IL-6) and eight (IL-8). Surprisingly, however, despite these interesting findings among the centenarians, this group did not find significant differences in microbiota composition between the younger adults and a group of older adults with an average age of 70 years.

**Table 1:** Studies investigating aging and the gut microbiome humans

Study	*N*	Population	Age	Age-affected microbiota	Age effect	Additional details
Hopkins et al. (2002) [130]	15	British	21–34 years 67-88 years 67–73 years CDAD	Bacteroides species diversity Bifidobacteria species diversity	**↑** **↓**	CDAD patients had greater diversity lactobacilli/clostridia but reducted bacteriodes, prevotella, and bifidobacteria
Hayashi et al. (2003) [131]	6	Japanese	79–84 years^a^	Clostridium rRNA subcluster XIVa Bifidobacteria # Ruminoccoccus obeum	**↓**	
Woodmansey et al. (2004) [132]	28	British	19–35 years 67–75 years 73–101 years HE	Bacterioides Bifidobacteria (no. and diversity)	**↓**	Hospitalized patients displayed age-related changes along with increased proteolytic bacteria no. and diversity
Van Tongeren et al. (2005) [74]	23	Dutch	70–100 years	N/A	N/A	High frailty scores associated with reduced lactobacilli, bacteroides, prevotella no.; increased enterobacteria
Biagi et al. (2010) [70]	84	Italian	25–40 years 59–78 years 99–104 years	Clostridium cluster XIVa Bacilli Proteobacteria	**↓** **↑** **↑**	Young and elderly showed similar microbiota, while differences observed in centenarians only.
Claesson et al. (2011) [72]	170	Irish	28–46 years >65 years	Firmicutes Clostridium cluster IV Ruminococcaceae	**↓** **↑** **↑**	
Claesson et al. (2012) [73]	191	Irish	28–46 years 64–102 years	N/A	N/A	Microbiota composition clustered by diet and residence location + significantly correlated with frailty, co-morbidity, and inflammation
Rampelli et al. (2013) [71]	9	Italian	38–102 years	Bacterial DNA gene expression for: short-chain fatty acid production Proteolytic functions Pathobionts	**↓** **↑** **↑**	
Jeffery et al. (2016) [40]	371	Irish	64–102 years	N/A	N/A	Longitudinal samples revealed temporal instability of microbiota Low microbial diversity associated with greater temporal instability Long-term care stays and antibiotic use associated with increased alterations in microbial composition and diversity
Jackson et al. (2016) [41]	1008	British Irish	42–102 years	N/A	N/A	Robust associations between frailty and gut microbiota *F. prausnitzii* negatively associated *E. dolichum* and *E. lenta* positively associated
Odamaki et al. (2016) [133]	367	Japanese	0–104 years	Proteobacteria/Bacteroidetes Firmicutes/Actinobacteria	**↓** **↑**	
Cattaneo et al. (2017) [42]	83	Italian	Mean ~ 70 years^b^	N/A	N/A	Gut microbial populations and peripheral inflammatory cytokines associated with cognitive impairment and brain amyloidosis

*CDAD* clostridium difficile-associated diarrhea, ^a^Age-effect compared to prior study of young adults; *HE* hospitalized elderly patients on antibotics, ^b^range and overall mean for the full study not reported (reported by study group)

In contrast, findings from the Irish ELDERMET cohort [72] did indicate alterations in the core microbiota of persons over 65 years of age. These changes were generally characterized by a greater proportion of *Bacteroides* spp. and distinct abundance patterns of *Clostridium* groups compared to younger individuals. Older individuals also displayed a loss of diversity-associated taxa, including Prevotella and associated genera [40, 73], which can contribute to instability in the microbiome composition. However, the authors noted that the variability in the microbiota profiles of the older persons was quite large—making phenotype prediction difficult. Several key factors—particularly diet and the use of antibiotics—were key predictors of these changes in both community-dwelling seniors and residents of long-term care facilities. These factors, diet in particular, could potentially explain differences between findings from the Italian and Irish cohorts. As such, these and other relevant factors necessitate the continued study of the microbiome across populations.

Notably, data from the ELDERMET cohort revealed an interesting potential association with physical frailty as evidenced by differences among older persons living in long-term care and/or rehabilitation facilities compared to community-dwelling peers [73]. These differences were also associated with several systemic markers of inflammation including IL-6, IL-8, CRP, and TNF-α. The association with frailty in the cohort was recently demonstrated more formally along with concordant findings from 728 female twins enrolled in the Healthy Ageing Twin Study [41]. These findings were similar to those from a prior small cohort of older adults from The Netherlands [74]. Thus, available data suggest that the gut microbiome may play at least some role in the development of physical frailty among the elderly.

Similarly, early findings now suggest that gut dysbiosis may contribute to age-related declines in cognitive function. Cattaneo et al. recently reported that brain amyloidosis and peripheral inflammation among cognitively impaired elders was associated with the abundance of pro- and anti-inflammatory gut microbiota [42]. Indeed, brain amyloid content and circulating inflammatory analytes were positively associated with the inflammatory bacteria taxon *Escherichia/Shigella* and negatively associated with the anti-inflammatory *E. rectale* taxon. To my knowledge, these are the first data directly linking the gut microbiota to age-related cognitive decline, though numerous studies in other models (e.g., Parkinson’s, multiple sclerosis, animal models of Alzheimer’s disease) exist to indicate a well-defined gut-brain axis which could contribute to age-related dementias [62, 75, 76]. Given the dramatic public health implications of understanding and intervening upon age-related dementias, continued research in this area seems highly warranted.

## Etiology of age-related changes in the microbiome

The precise etiologic explanation for these age-related changes remains incomplete. Across the age-spectrum, dramatic increases in the use of antibiotics and increasing pervasiveness of a high-saturated fat and high-sugar “western” diet are proposed to directly contribute to the depletion of important beneficial components of the microbiome [50, 77]. In turn, these changes contribute to chronic activation of the immune system and a dramatic rise in the prevalence of chronic inflammatory disorders. These two factors also represent key modifiable health factors which may contribute to exacerbated dysbiosis among older adults.

In the United States of America, rates of antibiotic prescription actually dropped from 2000–2010 among children and young to middle-aged adults [78]. In contrast, prescription rates increased among older adults with the most dramatic increases seen among persons ≥80 years of age [78]. Additionally, rates of antibiotic prescription have risen substantially in recent years in residential care facilities [79]. These trends may at least partially explain age-related changes in the microbiome, particularly those observed in the ELDERMET cohort among residents of long-term care facilities.

Similarly, age-related changes in nutrient intake may also contribute to late-life dysbiosis. It is well-recognized that diet is one of the primary contributors to gut health. Advanced age is associated with deterioration in various aspects of nutrient intake and absorption including dentition, salivary function, digestion, and intestinal transit time [73, 80]. Sensory changes, including taste and smell, may also alter the appetite making certain foods unappealing and thus altering eating habits [81]. It is possible that these changes contribute to dysbiosis though it may be more likely that altered immune responses to “inflammatory” foods among older adults may exacerbate microbial changes.

Another prominent possibility is that chronic activation of the innate and adaptive immune systems due to immunosenescence contributes to an altered bacterial composition in the gut. Such an affect may manifest at least partially due to known increases in hypothalamus-pituitary-adrenal (HPA) axis activity in advanced age [82] as HPA-mediated inflammatory stress responses are known to induce to both immune dysregulation [83] and dysbiosis [84]. Conversely, however, it remains possible—given the known inflammatory effects of dysbiosis—that changes to the microbiome due to other factors (e.g., diet) exacerbate inflammation and altered immunity. Thus, it is presently difficult to decipher the temporal relationship between these changes. Furthermore, this known interplay suggests a tantalizing hypothesis that the processes of immunosenescence and dysbiosis may in fact be interdependent.

Moreover, physical changes to the intestinal epithelial barrier may play a role in dysbiosis and age-related inflammation. Jakobsson et al. previously demonstrated that the composition of the gut microbiota is directly related to the leaky gut [85]. Recent evidence suggests now that intestinal permeability may increase with age. Man et al. [86] recently demonstrated that, compared to younger adults, ileal tissues from older adults demonstrated increased IL-6 concentrations that were accompanied by increased intestinal permeability as a result of elevated claudin-2. These data provide novel evidence from humans indicating the likelihood of a “leaky gut” during advanced age whereby the intestinal barrier preventing harmful substances from reaching the bloodstream is permeated. The leaky gut is well-associated with inflammatory bowel conditions but is now being proposed as a contributor to a wide variety of health conditions [87, 88]—with particular interest in a gut-brain axis which regulates the blood brain barrier [61, 62, 75, 76].

Though somewhat speculative at present, it is possible that the leaky gut is a primary source of inflammation long-observed within the circulation. Studies in *Drosophila* have reported that age-related changes in the microbiome increase intestinal permeability [89] and drive chronic inflammation [90]. More recently, Thevaranjan et al. [91] were the first to our knowledge to publish work from mammals directly supporting this hypothesis. Using germ-free and conventionally raised mice, this group reported that the germ-free animals did not display an age-related increase in systemic pro-inflammatory cytokines. Moreover, co-housing germ-free with old—but not young—conventionally raised mice increased circulating pro-inflammatory cytokines [91]. Anti-TNF therapy also reversed age-related microbial changes. These data are the strongest to date suggesting the critical role of gut changes in driving age-related inflammation and provide a solid backdrop for continued investigation in this area.

## Potential intervention strategies

Though additional research is certainly needed in this area, the aforementioned data do suggest that interventions designed to target the gut microbiome may be capable of producing beneficial effects on age-related inflammation and overall health. To date, research on such interventions is as limited as that on the aging microbiome in general. Still, within this small evidence base are some intriguing and promising findings that support the concept of intervening upon the microbiome.

Interestingly, some of the most promising strategies are not truly “gut-specific” but rather well-known interventions which are increasingly recognized to have beneficial effects on the gut microbiome. For instance, dietary habits appear to be one of the biggest drivers of gut health and microbiota composition [92–94]. Specifically, adherence to a high-fat diet commonly seen in Western cultures appears to be one of the driving factors in gut dysbiosis [95, 96]. Accordingly, interventions such as caloric restriction and/or implementing a Mediterranean-style diet may hold promise for balancing the gut microbiome [97, 98]. Moreover, studies exist to suggest potential benefits to specific dietary choices and/or nutritional supplements including (but not necessarily limited to) coffee [99], resveratrol [100], quercetin [100], and/or other polyphenol compounds [101]. Interestingly, many of these strategies have previously been purported to have anti-inflammatory and/or healthy aging properties. These new data suggest that perhaps influences at the level of the gut may be at least partially responsible for these purported benefits. However, studies are needed that specifically evaluate the influence of dietary changes on the gut microbiome and other health parameters among older adults.

Similarly, physical exercise is a clinically recommended intervention with well-established health benefits which is now purported to have important benefits on the gut [102–105]. Recent studies in laboratory animals have demonstrated that aerobic exercise provides a variety of beneficial effects on the gut including enhancing epithelial membrane integrity [106], increasing microbial diversity [106–109], and attenuating intestinal inflammation [106, 110, 111]. However, few human studies in this area have been published to date. Recently, Clarke et al. [112] recently reported that professional athletes (rugby players) displayed a higher diversity of gut micro-organisms compared to age-matched controls. However, extensive additional findings from humans—particularly in the form of longitudinal intervention studies—are needed to confirm these beneficial effects. Moreover, to my knowledge, no studies (human or animal) have evaluated the influence of exercise on the gut microbiome in late life. Additionally, questions remain regarding the effects of exercise modality and if these observed effects are specific to aerobic exercise compared to resistance training.

Perhaps not surprisingly, dietary supplementation with probiotics also appears to be among the most promising interventions for re-balancing the gut microbiome. Well-known actions of probiotics include antimicrobial activity, enhancement of intestinal barrier function, and immunomodulation via actions on a wide variety of immune cells [113]. In line with these established mechanisms, studies among older adults have demonstrated beneficial effects of probiotic preparations on gut microflora composition [114–116] and systemic immunity [116–118]. van Beek et al. also recently reported that 10 weeks of supplementation with the probiotic *Lactobacillus plantarum* WCFS1 prevented age-related decline in the colon mucus barrier in a mouse model of accelerated aging [119]. Probiotics have also been proposed to potentially have anti-inflammatory properties [113]. Such an effect seems plausible given the effects of probiotics on the immune system. To date, several studies have investigated this potential anti-inflammatory affect with the overall effect appearing to be modest [116, 118–122]—though more definitive research in this area is certainly warranted.

More research is also needed regarding the ability to utilize probiotics as a vehicle to deliver other therapeutic compounds. Indeed, genetically modified probiotics have been purported as highly promising treatment strategies [123–125] as they offer a potentially efficacious method to deliver drugs or other therapeutic proteins with precision and a higher degree of site specificity than conventional drug regimens [126]. For instance, Steidler et al. previously reported on the utility of *Lactococcus lactis* as a vehicle for delivering IL-10 for the treatment of inflammatory bowel disease [127]. Elsewhere this strategy has been proposed as a potential method of delivering antihypertensive therapeutics [128, 129]. Given the relative infancy of this field, however, the overall body of literature in this area is sparse. Future studies are certainly warranted on the potential utility of genetically modified probiotics in the treatment of age-related inflammation and associated diseases.

## Conclusions

**Fig. 3:**
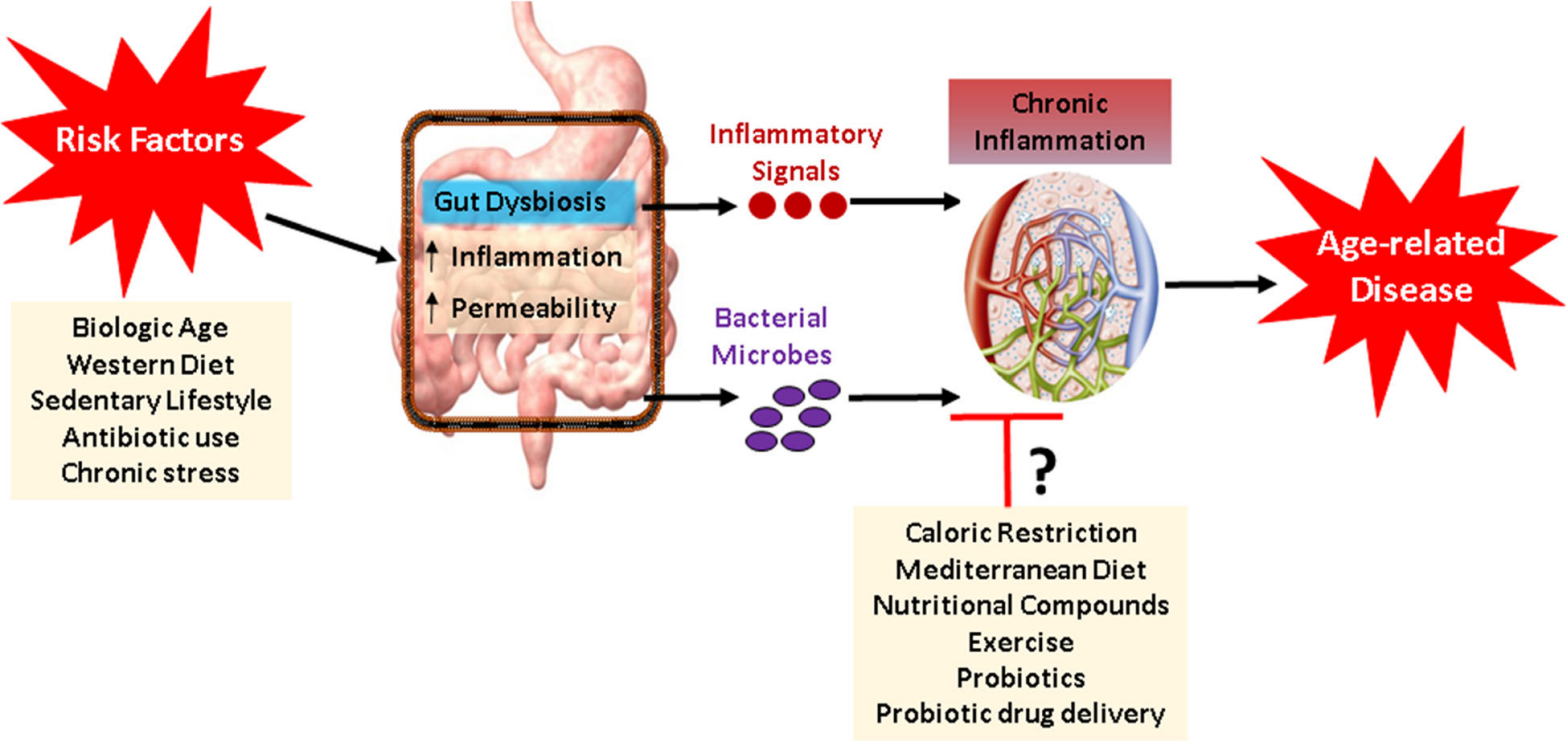
Simplified schematic outlining the potential relationships among health risk factors, gut dysbiosis, inflammation, and age-related disease—as well as potential interventions for attenuating gut-associated inflammation

As the population ages across developed nations worldwide, the need for healthcare solutions to ease the burden of age-related diseases grows. Treatments for chronic inflammation represent a particularly promising strategy given the observation of inflammation in “healthy aging” as well as nearly every age-related disorder or disease. Growing evidence indicates that the gut microbiome may represent a novel site of intervention for the prevention and/or treatment of late-life inflammation. A variety of biologic, medical, and lifestyle factors appear to contribute to gut dysbiosis in late-life, and interventions specifically designed to target these factors may be useful in restoring microbial balance and attenuating inflammation (Fig. 3). Still, much remains to be unraveled as it relates to the influence of the gut microbiome on age-related inflammation—particularly as it relates to the potential efficacy of gut-directed interventions. Thus, this field appears to offer great opportunities for aging-related research. I look forward to continued investigation in this area as it has the potential to yield tremendous breakthroughs for improving the health and quality of life of older persons.

## Abbreviations

CRP: C-reactive protein
HPA: Hypothalamus-pituitary-adrenal
IL-6: Interleukin 6
IL-8: Interleukin 8
SCFA: Short-chain fatty acids
TLR: Toll-like receptor
TNF-α: Tumor necrosis factor alpha
